# The impact of resilience on academic performance with a focus on mature learners

**DOI:** 10.1186/s12909-024-06099-2

**Published:** 2024-10-07

**Authors:** Alexandra Steel, Nilushi Karunaratne, Betty Exintaris, Simon James, Abdullah Al-Juhaishi, Angelo Don, David Wei Dai, Angelina Lim

**Affiliations:** 1 Faculty of Pharmacy and Pharmaceutical Sciences, Monash University, 381 Royal Parade, Parkville, Melbourne, VIC 3052 Australia; 2 School of Information Technology, Deakin University, Geelong, VIC Australia; 3 UCL Institute of Education, University College London, London, WC1H 0AL UK; 4 Murdoch Childrens Research Institute, Royal Children’s Hospital, Melbourne, VIC Australia

**Keywords:** Self-regulated Learning, Mature Age Learner, School Leaver

## Abstract

**Background:**

Resilience is an essential psychological trait that empowers individuals to adapt and thrive in the face of challenges. Although it is acknowledged that health professionals need to possess high levels of resilience, there has been limited research comparing how different groups of health students, particularly school leaver undergraduates and mature age graduate entry students, develop resilience in their coursework.

**Methods:**

This study combines both objective (academic grades with validated survey results) and subjective data (interviews) to compare how resilience is related to academic coursework performance for two groups of pharmacy students: the mature age graduate entry (GE, *N* = 64) learners and school leaver undergraduate (UG, *N* = 208) learners. We employed a sequential explanatory mixed methods design using surveys, academic performance data and semi-structured interviews. The survey tapped constructs related to resilience (burnout, stressors and coping methods) while the interviews elicited a more nuanced understanding of individual and environmental factors.

**Results:**

Although there was no statistical difference in burnout experience between the two groups, GE students exhibited more positive resilience attitudes than UG when selecting resilience statements on the survey. Both cohorts indicated in the survey that engaging in distraction activities (physical exercise, sleeping, listening to music, anything other than the stressor) was their most preferred method of relieving stress. Within UG student survey responses, those who indicated support from partners, friends and family had better academic performance, while those who did not report coping methods did worse academically. The three key environmental factors we identified that contributed to both undergraduate and graduate entry resilience were workload, feedback provision and psychosocial support.

**Conclusions:**

Currently, there is still a need for resilience programs geared at academic success to be implemented in higher education. This study provides objective evidence of academic success coupled with exploration into the nuances of resilience amongst different student groups. It not only highlights the differing resilience development strategies and burnout coping mechanisms in emerging health professionals, but showcases the juxtaposition of two different learner groups (UG and GE students) within a discipline. The cross-cohort facilitation of learning as in the discipline-specific strategies identified can help both groups develop resilience and inform future innovations. By comparing mature-age graduate students and younger-in-age undergraduate students, we identified a wider range of strategies and more positive attitudes to burnout in mature-age students. Health and clinical educators in university health degrees, clinical placements and clinical workplaces can develop effective training materials based on findings from this study to 1) assist undergraduate younger-age health students with developing resilience and 2) further refine mature-age health students’ and practicing health professionals’ resilience in today’s fast-paced clinical workplaces.

**Supplementary Information:**

The online version contains supplementary material available at 10.1186/s12909-024-06099-2.

## Background

Resilience, an essential psychological trait, empowers individuals to adapt and thrive in the face of challenges, bolstering mental health and overall well-being [1]. Understanding resilience becomes increasingly vital in today's fast-paced world, where mental well-being is recognized as integral to overall health [2]. This is particularly pertinent in tertiary learning, especially within the health professional discipline, where the ability to navigate challenges and setbacks is essential for lifelong learners [3]. Within the health professional discipline, so far there have been limited cross-cohort analyses of resilience, which means differences in resilience strategies in student cohorts have been under-researched. There is an urgent need to address this gap since the tertiary education space is fast changing, with many mature learners joining the undergraduate curriculum with a cohort of high school leavers, making teaching and assessment design more challenging to maintain academic equity [4]. Within health care education, students encounter a rigorous academic environment marked by complex coursework and demanding exams, leading to heightened stress levels [5–7]. This stress can have profound effects, impacting not only academic performance but also overall health and quality of life [1]. Understanding how this stress impacts different types of students like graduate entry (GE) and undergraduate (UG) students, as well as their coping strategies, can help assist academics with future interventions and support.

Resilience in this context encompasses more than just overcoming obstacles; it involves cultivating the ability to thrive amid academic challenges and promoting personal growth and adaptability [8]. In this study, we employed the cognitive-behavioural framework of resilience due to its robust theoretical foundation, relevance to education, and holistic approach to compare resilience levels between GE and UG learners [6]. This framework delves into cognitive processes concerning stress and problem-solving skills, revealing how learners perceive and handle stressors differently. For example, GE learners may leverage life experiences and coping mechanisms acquired over time to overcome obstacles. Specifically, we applied this framework by incorporating a series of Likert statements into our surveys, designed to measure cognitive and behavioral responses to stress. These statements included items such as "I would try to think of new solutions" and "I would use my past successes to help motivate myself," which directly relate to the cognitive-behavioral processes outlined in the framework. This structured approach allowed a systematic assessment of how each group identifies, interprets, and manages academic challenges, providing a comprehensive understanding of their resilience levels.

Mature age learners often face unique challenges in educational settings, requiring a high degree of resilience to overcome obstacles and succeed academically. These learners may juggle multiple responsibilities such as work, family obligations, and financial commitments, which can add significant stressors to their educational journey [9]. Interventions targeting resilience often overlook the specific needs and experiences of different student demographics and provide a one-size-fits-all approach [9]. Additionally, societal stereotypes and stigma surrounding mature learners in educational settings can further exacerbate feelings of isolation and hinder access to support networks [9]. Conversely, GE learners may have developed stronger growth mindsets and learning strategies from a previous degree and are not disheartened as easily when receiving constructive feedback or poor marks [3, 10]. Therefore, insight into the underlying mechanisms driving resilience in each group will enable academics to develop targeted interventions to support diverse students.

Currently, there is a strong need for resilience programs geared at academic success to be implemented in higher education. To implement these interventions, we need to understand the nuances of the different learners in our cohorts. The exploration of UG and GE students' resilience has not been widely tackled. This study therefore is designed to compare and contrast the resilience attitudes and behaviors between mature age (GE) learners and school leaver (UG) learners by combining both quantitative objective measures of academic success (grades) with qualitative data to provide valuable insights into how resilience impacts academic performance. We specifically look into four different assessment types that are common to health professional students. This approach will provide robust evidence and identify specific needs and effective strategies for each student group to inform the development of tailored support systems to enhance resilience and academic success in diverse cohorts of healthcare students.

## Methods

### Context

Our institution offers a pharmacy curriculum with a GE pathway [11] and provides an opportunity for individuals who have completed a relevant science-related degree program to enter our four year Bachelor’s Degree at the start of year three. To be eligible for this pathway, students must be postgraduate students (mature learners) who have completed a prior science related discipline and achieved a distinction average for their overall grade. To catch up with the general cohort, these students complete a six week intensive bridging course to consolidate missed content in the first two years of the degree. During this intensive bridging course, GE students are delivered the same content as the UG students from first to second year at an accelerated pace and undergo the same assessments.

In order to compare the resilience strategies adopted by the UG and GE cohorts to facilitate cross-cohort learning, in this study we collected resilience strategies from a validated survey tool derived from the Motivated Strategies for Learning Questionnaire (MSLQ) [12, 13], and correlated survey responses with both cohorts’ academic assessment results to understand if students’ resilience strategies impacted their academic performance. More specifically, we examined four different types of assessments (multiple choice quiz, an oral simulation exam, a practical exam and a written exam) which are common assessments in health professional education. We also further unpacked the nuances of the resilience strategies and burnout between the groups with semi-structured interviews within a sample of the participants.

### Description of assessments delivered to UG and GE cohorts

Both UG and GE cohorts completed four assessments (please see below) within the first year of entering our degree. These four different assessments test different skills articulated in Bloom's taxonomy; the assessments for UG and GE cohorts were identical.

#### Calculations (Multiple choice question)

This assessment was a 20 question multiple choice quiz which consisted of questions on pharmaceutical calculations which they studied via online modules prior to the assessment.

#### Objective Structured Clinical Examination (OSCE) (Oral simulation exam)

*OSCEs* consisted of a three station assessment where each station required the student to manage a standardized patient. Students were tested on common minor ailments e.g. Diarrhoea, Constipation.

#### MyDispense (Practical examination)

This was a practical examination that was assessed through a virtual software designed by our institution called MyDispense [14] which simulated a community pharmacy. Students were required to dispense four prescriptions and were marked on their accuracy of dispensing.

#### Final examination (Written)

The final examination was a 12 question written exam requiring short answer responses testing clinical and scientific knowledge. This exam was out of 100 marks.

### Study design

This study was guided by pragmatism and employed a sequential explanatory mixed methods approach. This study was approved by the Institute’s Human Research Ethics Committee (Project ID 40429).

### Participants and setting

All participants were students who were enrolled in a tertiary institution in Melbourne, Australia in 2023.

### Outcomes

The primary outcome of this study was to identify differences in resilience strategies using a validated survey tool derived from the MSLQ [12, 13], comparing responses from UG and GE cohorts and how certain questions impacted on different types of assessments. Secondary outcomes included understanding what environmental and individual factors impacted these learners.

### Data collection and integration

Assessment grades were exported directly from the institute’s assessment platform. A resilience survey (See Additional File 1) was given to all UG and GE students at the end of their first semester. The questionnaire consisted of six questions. Question one was based on a five point Likert scale and derived from the MSLQ [12, 13], which is one of the most widely used instruments to measure students' motivation and self-regulated learning [15] while Questions 2–6 were open-ended. To integrate the quantitative and qualitative data, we followed the data integration steps mapped out for sequential mixed methods design by Ivankova et al. [16]. We designed the interview guide (See Additional File 2) based on which factors were seen to impact the differences between groups, and factors were seen to influence resilience. We also asked participants to expand on their individual survey responses. The development of the interview guide was an iterative process which involved all team members, and the guide was revised after the first nine interviews with the team meeting after every three interviews to discuss changes. Each interview 20–30 min in duration and conducted over Zoom by thesame facilitator.

### Data analysis

All quantitative data analysis was performed using R [17] and the base statistical package. To assess the primary outcome of differences in resilience strategies between UG and GE cohorts and how they impacted on different types of assessments, we used correlation analysis. Open responses from the resilience survey were coded using content analysis to fixed categories. These categories were then compared with assessment grades alongside the fixed Likert scale survey responses. Results from the different forms of assessment generally were not normally distributed (with the exception of the exam scores) and hence the median and interquartile range were used to summarise overall results. We used t-tests to compare student groups with respect to exam scores and the standard two-proportion test to compare percentages. The Wilcox rank sum test with continuity correction was used to compare groups with respect to the remaining assessment scores, along with Kendall’s tau correlation coefficient for ordinal responses from the survey.. A significant finding for the t-test indicates that the means of the two groups differ, while for the two-sample Wilcox test it suggests that a randomly chosen individual from one group is more likely to be higher/lower than a randomly chosen individual from the other. This is similar for Kendall’s tau, which tallies the proportion of pairs where increasing one (possibly ordinal) variable also results in an increase/decrease to the other. It ranges between -1 and 1 depending on the effect direction. Fisher’s test was used in cases of low numbers. A *p*-value less than 0.05 was considered statistically significant.

For the secondary outcome of understanding the environmental and individual factors impacting these learners, thematic analysis was used to code the interview data. NVivo (QSR International, Version 1.3) was used to manage the coding. A thematic analysis approach was used to analyze the data, which involved identifying patterns and themes in the data [18]. The analysis was carried out by three investigators (AS, NK, BE) who initially read the first four interview transcripts independently and then met to discuss and generate an initial codebook [19]. In the second round, two researchers (NK, AS) coded two interviews independently using the initial codebook, and then compared and discussed their findings to arrive at a consensus for a final codebook (See additional file 3). A third researcher (BE) was present at the discussion to remediate any disagreements. This round of coding and discussion resulted in achieving a Cohen's kappa for intercoder reliability of 0.81 between NK and AS’s coding, signifying strong agreement between coders [20]. The remaining interviews were coded by one researcher (AS) using the final codebook.

### Team reflexivity

At the first phase of coding, the team conducted a team reflexivity exercise by discussing what the definition of resilience isAll authors agreed that resilience was the ability to respond, recover and cope with setbacks and adversity. Time management and managing external distractors to reduce risk of burnout were also discussed.

## Results

All of the 64 GE students and 208 UG students (74%) completed the self-reported resilience survey (See additional file 1). On average, GE students were 3.2 years older than the UG students. In the GE cohort of participants, 44% self-reported as male, 56% as female and two students were international students. In the UG cohort, 33% self-reported as male, 67% as female and four students were international students.

### Quantitative results—objective measures of academic success related to resilience survey responses

We first investigated the academic outcomes of both groups across the same assessments. We then investigated if there was any relationship between students’ responses to the different statements in the validated resilience survey derived from the Motivated Strategies for Learning Questionnaire (MSLQ) [12, 13] and their academic grades for different assessment types. Table 1 summarises the overall results for each of the assessments for the two cohorts, reporting the median and IQR as well as the pass rates. A statistical difference (using the Wilcox rank sum test) was observed for the distributions of exam scores (*p* < 0.001) while in terms of pass rates, statistically significant differences (using Fisher’s test due to low failure numbers for the GE students) were observed for MyDispense (*p* < 0.001), OSCE (*p* < 0.001) and the final exam (*p* = 0.026).

**Table 1:** Median [IQR] for students who passed along with pass/participation (%)

	**Calcs (mcq)**	**Mydispense (practical)**	**OSCE (oral)**	**Final exam (written)**
**1st years who completed the survey (school leavers) (*N* = 196)**	93.3 [13.3] *P* = 173/194 (89.2%)	95 [5.4] *P* = 151/196 (77.0%)	91.4 [9.4] *P* = 170/196 (86.7%)	69 [14.5] *P* = 169/192 (88.0%)
**1st years whole cohort (school leavers) (*N* = 301)**	93.3 [13.3] *P* = 257/297 (86.5%)	94.6 [5.9] *P* = 209/281 (74.4%)	90.6 [10.7] *P* = 232/279 (83.2%)	68.5 [15.0] *P* = 243/278 (87.4%)
**BTP (graduate entry) who completed the survey (*N* = 64 – all)**	95 [10] *P* = 51/64 (79.7%)	93.1 [7.4] *P* = 60/64 (93.8%)	86.2 [9.8] *P* = 64/64 (100%) *p* < 0.001 fisher < 0.001	75 [20.5] *P* = 62/64 (96.7%) *p* = 0.048 fisher = 0.026

One question in the survey prompted students with the scenario: “You received an N on your most recent assessment. The grades for your other two assessments were also lower than what you wanted.” and then asked them to respond on a Likert scale across a number of statements regarding how they would feel or what they might do if they found themselves in this position. The responses are summarised in Table 2. Results are similar for many questions, although overall GE students tended to exhibit more positive resilience attitudes than UG. In particular, there were two statements where UG and GE cohorts differed with regard to statistical significance. Undergraduates were more likely to agree with the statement “I would feel like everything was ruined or going wrong” with 37.1% selecting agree and 17.4% strongly agree in UG compared with 17.9% selecting agree and 14.3% strongly agree in the GE cohort (*p* = 0.0015 using Kendall’s tau test). Graduate entry students were then more likely to agree with the statement “I would use my past successes to help motivate myself” with 53.7% agreeing and 18.5% strongly agreeing compared with 31.6% and 14.6% respectively in UG (*p* = 0.0149).

**Table 2:** Summary of Responses for Undergraduate (UG) and Graduate entry (GE) according to survey questions

**Statement**		Strongly Disagree	**Disagree**	**Neutral**	**Agree**	**Strongly Agree**	**No Response**
I would begin to doubt my chances of success in the course	UG	2 (1.2)	18 (10.8)	25 (15)	82 (49.1)	29 (17.4)	40 (24)
	GE	4 (7.7)	8 (15.4)	11 (21.2)	20 (38.5)	12 (23.1)	9 (17.3)
I would be disappointed	UG	1 (0.9)	4 (3.5)	5 (4.4)	63 (55.8)	83 (73.5)	40 (35.4)
	GE	0 (0)	2 (5.6)	3 (8.3)	22 (61.1)	28 (77.8)	9 (25)
I would begin to think my chances of getting the job were poor	UG	11 (6.2)	48 (27.1)	36 (20.3)	42 (23.7)	19 (10.7)	40 (22.6)
	GE	4 (6.9)	23 (39.7)	11 (19)	11 (19)	6 (10.3)	9 (15.5)
I would feel like everything was ruined and going wrong	UG	6 (3.6)	27 (16.2)	31 (18.6)	62 (37.1)	29 (17.4)	41 (24.6)
	GE	6 (10.7)	18 (32.1)	13 (23.2)	10 (17.9)	8 (14.3)	9 (16.1)
I would try to think of new solutions	UG	2 (1.3)	6 (3.8)	20 (12.7)	88 (56.1)	39 (24.8)	41 (26.1)
	GE	0 (0)	3 (6.4)	5 (10.6)	29 (61.7)	17 (36.2)	10 (21.3)
I would use my past successes to help motivate myself	UG	6 (3.5)	27 (15.8)	43 (25.1)	54 (31.6)	25 (14.6)	41 (24)
	GE	3 (5.6)	0 (0)	12 (22.2)	29 (53.7)	10 (18.5)	10 (18.5)
I would set my own goals for achievements	UG	2 (1.2)	11 (6.7)	29 (17.6)	82 (49.7)	31 (18.8)	41 (24.8)
	GE	1 (2)	1 (2)	8 (15.7)	31 (60.8)	13 (25.5)	10 (19.6)
I would seek encouragement from my family and friends	UG	13 (8.3)	31 (19.7)	28 (17.8)	44 (28)	39 (24.8)	41 (26.1)
	GE	1 (2)	6 (12)	10 (20)	23 (46)	14 (28)	10 (20)
I would try to think about my strengths and weaknesses to help me work better	UG	1 (0.6)	11 (6.8)	26 (16.1)	82 (50.9)	35 (21.7)	41 (25.5)
	GE	0 (0)	3 (5.9)	10 (19.6)	28 (54.9)	13 (25.5)	10 (19.6)
I would see the situation as a challenge	UG	2 (1.2)	19 (11)	36 (20.9)	74 (43)	24 (14)	41 (23.8)
	GE	0 (0)	5 (9.3)	11 (20.4)	27 (50)	10 (18.5)	11 (20.4)
I would do my best to stop thinking negatively	UG	5 (2.9)	17 (9.8)	41 (23.7)	69 (39.9)	23 (13.3)	41 (23.7)
	GE	3 (5.3)	3 (5.3)	11 (19.3)	29 (50.9)	7 (12.3)	11 (19.3)
I would see the situation as temporary	UG	3 (1.7)	20 (11.5)	33 (19)	77 (44.3)	22 (12.6)	41 (23.6)
	GE	3 (5.4)	3 (5.4)	11 (19.6)	28 (50)	8 (14.3)	11 (19.6)
I would just give up	UG	57 (29.5)	60 (31.1)	24 (12.4)	11 (5.7)	3 (1.6)	41 (21.2)
	GE	19 (30.2)	23 (36.5)	8 (12.7)	2 (3.2)	1 (1.6)	11 (17.5)
I would change my career plans	UG	49 (25.8)	45 (23.7)	38 (20)	16 (8.4)	6 (3.2)	42 (22.1)
	GE	17 (27)	25 (39.7)	7 (11.1)	2 (3.2)	1 (1.6)	12 (19)
I would not change my long term goals and ambitions	UG	6 (3.7)	7 (4.3)	36 (22.1)	69 (42.3)	33 (20.2)	45 (27.6)
	GE	1 (2)	7 (14)	8 (16)	20 (40)	14 (28)	14 (28)

For each of the statements in this question, and for each cohort, we also looked at whether responses to any questions were predictors of performance on the four assessments using correlational analysis. With 15 statements, 2 cohorts and 4 assessments, it is probable that some results of statistical significance will arise by chance, however we can make note of some interesting results where both a significant result was observed (*p* < 0.05) and the effect size was above 0.2 or below -0.2.

• Graduate entry students who agreed with the statement “I would be disappointed” were *less* likely to do well on the MCQ test (*p* = 0.0184, tau = -0.2775);
• Graduate entry students who agreed with the statement “I would seek encouragement from my family and friends” were more likely to do well on the MyDispense exam (*p* = 0.0293, tau = 0.2287);
• Graduate entry students who agreed with the statement “I would just give up” were less likely to do well in the OSCE (*p* = 0.0029, tau = -0.3198),
• Graduate entry students that agreed with the statement “I would change my career plans” were less likely to do well in the OSCE (*p* = 0.0046, tau = -0.3076) and also less likely to do well in the exam (*p* = 0.0455, tau = -0.2194).

On the whole, other significant results with lower absolute values of tau aligned with what might be expected in terms of positive/negative resilience behaviors and attitudes, however there were two questions which showed a counter-intuitive result.

• Undergraduate students who responded “I would be disappointed” were somewhat *more* likely to do well on the OSCE (*p* = 0.042, tau = 0.132);
• Undergraduate students who responded “I would try to think about my strengths and weaknesses to help me work better” were somewhat *less* likely to do well both on the MCQ (*p* = 0.0294, tau = -0.1494) and the exam (*p* = 0.0201, tau = -0.1452).

This analysis therefore supports the argument that positive resilience behaviors and attitudes are positively correlated with success in assessment.

Furthermore, students were asked whether they have experienced burnout and to elaborate on how they cope, as well as what causes burnout. Table 3 summarises the overall percentages of each cohort who reported as having experienced burnout (no statistical difference was observed between cohorts using a two proportion test, *p* = 0.8787).

**Table 3:** Students self-reporting as having experienced burnout

**Cohort**	**Yes**	**No**	**No Response**
UG	118 (84.3)	22 (15.7)	56 (28.6)
GE	36 (81.8)	8 (18.2)	20 (31.3)

Tables 4 and 5 categorises and shows percentages for general stressors and relief of stressors across UG and GE students.

**Table 4:** General stressors

**Theme**	**UG**	**GE**
Fear of academic failure through formative/non-formative performance markers (presentations, fear of number of exams or assessments and/or failing them, compared to with friends who perform better)	60 (43.2)	9 (20)
Increased workload (academic, hurdle assessments, non-academic, difficulty understanding content, expectations, duties, forgetting deadlines, falling behind, not employing time management)	82 (59)	29 (64.4)
Extraneous circumstances (family, social interactions, relationships, work/life balance, money, work, societal unfair treatment, negative interactions such as being yelled at, things that are outside of your control/comfort, uncertainty in life, being late to class)	17 (12.2)	1 (2.2)
Racism	1 (0.7)	
Natural Stress		3 (6.7)
No specific information	3 (2.2)	5 (11.1)

**Table 5:** Relief of stressors

	UG	GE
Support from partner, friends and family	20 (14.4)	10 (22.2)
Engaging in a distraction (physical exercise, movie, reading, watching funny videos, sleeping, hobby, eating good food, listening to music, watching the sunset, anything other than stressor)	65 (46.8)	24 (53.3)
Proactive management of life (time management, organising of daily duties and upcoming assessments, working through the task in a timely manner without procrastination)	42 (30.2)	16 (35.6)
Emotional modulation (crying, sitting and reflecting, breathing exercise, moody, silence, looking at things with alternate perspective, meditation, journalling, spa, looking forward, going to church)	21 (15.1)	1 (2.2)
No further information/No coping methods	21 (15.1)	6 (13.3)

We also asked students what the university could do to support resilience, what the major hindrances are and strategies for the future (Tables 1, 2 and 3 respectively in Additional File 4). The most common response from UG students (35.2%) stated facilitating a supportive environment (support guidelines, after-school tutoring, return past papers to students, social events to break up studies, practice sessions before major assessments, adequate reminders, more engaging teaching associates, making hurdle assessments easier, resilience course, less work load, more time for break/study, spacing assessments), whilst the most common response from GE students (39.4%) suggested increased activities (hamburger feedback, time management courses, help sessions on unsure topics, counselling, teaching soft skills early in career, i.e. dealing with burnout, listening to pharmacist alumni, stress relief-dog). For undergraduates, students who mentioned family circumstances (37.6%) in elaborating on experiences of burnout were more likely to do well on the MyDispense test (median of 97.25 compared with 92.15 for those who made no mention, *p* = 0.0006). UGs who indicated proactive life management (30.2%) as a strategy to relieve stress tended to do slightly better on the exam (mean of 69.7 compared with 64.6 or medians 70.8 and 66.0 respectively, *p* = 0.0358 using a t-test). UGs who indicated the university could facilitate a more supportive environment (35.2%) tended to do slightly worse on the exam (mean of 61.3 compared with 68.4 or medians of 62.5 and 70.0 respectively, *p* = 0.0076 using a t-test). UG students who indicated no further info or that students should develop resilience on their own (but did not skip the question) (24.6%) performed slightly better in the exam (mean 70.8 compared with 64.3 or medians 72.0 and 65.5 respectively, *p* = 0.0467 using a t-test). Those who indicated proactive planning (57%) as one strategy to adopt in the future tended to perform better on the OSCE (median of 91.6 compared with 85.8, *p* = 0.0207 using a Wilcox test). UGs who indicated self-care (19.3%) as one strategy to adopt in the future tended to perform worse on the exam (mean of 59.3 compared with 67.4 or medians of 56.8 and 67.8 respectively, *p* = 0.0412 using a t-test).

For graduate entry students, students who indicated rest and recuperate as a strategy when elaborating on burnout (31.9%) tended to score worse in the MCQ (median of 76.0 compared with 90.8, *p* = 0.0064). GE students who noted fear of academic failure (20%) under general stressors performed worse on the MCQ test (median of 80.0 compared with 95.0, *p* = 0.0185). Those who noted increased activities (39.4%) in what the university can do did worse in the MCQ (median of 80 compared with 100, *p* = 0.0058).

### Context of resilience aligned with the cognitive-behavioural framework of resilience (Qualitative Data)

Having examined differences in resilience and academic success in the quantitative analysis, we then explored the environmental and individual factors that may have contributed to the differences. A full summary of key quotes is provided in Additional File 3.

#### Environmental factors

The three key environmental factors that contributed to both undergraduate and graduate entry resilience throughout the semi-structured interviews were workload, feedback provision and psychosocial support. Seven graduate entry participants discussed receiving feedback from their educators and what they did with that feedback, such as BTP007, who “look[s] at it as a way to improve in the future”. Similarly, UG0010 “like[s] feedback, because [they] always want to know what [they’re] doing wrong and how [they] can do it better next time.” Ten undergraduate students discussed receiving and acting on feedback.

Four graduate entry students highlighted their workload, including BTP003 stating that “it's …difficult with work and life balance sometimes.” Six undergraduate participants mentioned workload, with UG001 reporting that they “reduce [their] sleep and…usually [don’t] study on weekends, but these days [they] should study weekends as well, because there’s no time.” Finally, five graduate entry participants and five undergraduate participants discussed the influence of psychosocial support. This support came from various sources, such as BTP007 discussing how “practising with friends makes such a huge difference” and UG005 “did seek out help from [a] school advisor”.

#### Individual factors – active cognition, information processing biases and executive control

Amongst the UG participants, a single participant demonstrated active positive cognition, with a single participant also demonstrating dysfunctional cognition with regards to their pharmacy studies. A positive outcome reported by UG003 was that they “might not know everything, but [they] know at least a bit of everything, so [they] won’t be completely lost”. In contrast, UG009 detailed that “if it’s a really low score that [they] didn’t expect [they’ll] get very sad.” Maladaptive information processing biases were reported by two participants surrounding feedback. UG0012 stated that they would “avoid them as long as [they] could, because [they] feel like they are criticising [them] instead of like giving [them] feedback.” Contrasted to this, UG007 showed adaptive information processing bias as they “make [their] own assumptions based off of the rubric of what [they] needed to improve on”. Finally, adaptive executive control was demonstrated by four participants, including prioritisation techniques detailed by UG0010, such as “spending more…time and prioritising the ones [they] don’t understand rather than…trying to give each topic equal time.” Two participants reported maladaptive executive control, such as UG0013 who “thought [their] biggest weakness is less about study techniques and [is] getting [them]self to do stuff”.

In comparison, no graduate entry participants reported dysfunctional cognitions. Three graduate entry participants reported active positive cognitions, with BTP001 stating that they were “not perfect, but [they] don’t really have an issue with that.” Adaptive information processing biases were reported by one participant, BTP008 reporting that they “take [their] mistakes very seriously…it’ll still scar [them], which is good because [they] remember.” Maladaptive biases were reported by three participants that affected their studies, including BTP006 stating that they were “too nervous to like stuff up in front of [their peers] or like to make a fool of [themselves] if [they] didn’t know what [they] were doing.” Finally, five participants demonstrated adaptive executive control with regards to their studies. BTP002 reported that they “try to think about what [they’ll] do if tomorrow is [their] exam, like what [they] need to learn if [they] have to go to exam tomorrow when [they’re] really busy.” Only one participant, BTP009, discussed maladaptive executive control, stating that they “cut down the amount of time [they] spend on doing something which probably impacts on the quality of the way that [they’ve] done it.”

## Discussion

This paper aimed to evaluate the attitudes towards resilience and behaviours exhibited by UG and GE learners, with a focus on the differences between the two given the lack of a substantial body of research. By investigating the relationship of resilience and actual academic success across different assessment types, we generate insight for academics into how to better support different student cohorts (UG and GE) for certain types of assessment categories. The ability to overcome setbacks and navigate challenges is essential for longevity in a career in the healthcare environment [1]. The requirement for resilience in the healthcare space begins with health professional education, whereby students are subjected to deadlines, examinations and additional stressors [5]. With the diverse cohort of learners in our health professional degrees, comparing and contrasting the resilience attitudes and behaviours of mature age learners and UG learners can provide valuable insights for academic support strategies. A major strength of our paper is that patterns in self-reported resilience strategies are corroborated by differences in academic achievement as measured by objective assessment instruments. Quantitative results showed that GE students were more likely to exhibit positive resilience attitudes and behaviours, which aligns with existing findings that prior educational experiences that mature age learners have obtained could contribute to stronger growth mindset and learning strategies, thus positively impacting on their resilience [9, 10].

These additional education experiences may have influenced mature age learners to appreciate the difference between simply performing well on assessments, and the lifelong learning required to succeed in a career path such as healthcare. This could result in a more adaptive approach to receiving grades and place significance on these, when compared to UG students who may place more self-worth on their academic performance [6, 10]. The correlation between resilience statements and outcomes on assessments was mostly as expected for these GE students, and UG participants were less likely to report positive resilience statements. Without prior exposure to higher level education whereby a growth mindset is advantageous, undergraduate students could identify more strongly with a fixed mindset, resulting in grades significantly affecting their mindset and their ability to take feedback onboard and adapt as required for future assessments [10]. Although, the correlation between the majority of the UG participants’ statements and academic results may seem expected, this study shows insight into the resilience of different student groups and has the novelty of combining objective assessment data with subjective data to further support these findings. Our study therefore has generated objective evidence for future resilience educational innovations targeting resilience.

When looking at different assessment types, UG students who reported being disappointed were more likely to do well on the OSCE and those who used a positive resilience statement regarding using strengths and weaknesses to work better were less likely to do well on the MCQ assessment and the exam. The quantitative findings showed an overall positive correlation between appropriate and effective burnout management strategies and outcomes of assessments amongst UG participants. Strategies such as proactive life management, proactive planning and developing resilience on their own were positively correlated with the outcomes of assessments. Contrastingly, students who indicated that the university could facilitate a more supportive environment and use self-care were less likely to perform well. GE students reported rest and recuperation as a strategy for managing burnout; those who reported fear of academic failure as a stressor and those who listed more items that the university could do tended to perform worse on the MCQ assessment. Interestingly, overall, GE students also performed significantly better in three of their assessments when compared to UG students, including the Mydispense assessment; OSCEs; and final examinations. These results provide evidence to support a positive correlation between positive resilience attitudes and burnout management strategies and success in assessments.

The qualitative results further added to the findings by exploring both environmental and individual factors contributing to the resilience of UG and GE students, aligning with the cognitive-behavioural framework of resilience [6]. Both adaptive and maladaptive environmental factors were referenced by UG and GE participants. The three notable factors contributing to resilience included the impact of receiving feedback, the influence of their workload, and the positive correlation between psychosocial support and success. Both UG and GE students discussed the importance of feedback and how this guided their approach to future assessment tasks, suggesting potential for positive outcomes. However, it is important to consider the likelihood of students becoming disheartened by feedback, which is more likely to happen amongst undergraduate students given their lack of exposure to feedback in a higher education context [3, 10]. In the interviews, GE students highlighted the difficulty of balancing work and life commitments, which aligns with the prospect of mature age learners being more likely to have work, family and financial obligations [9]. Both UG and GE students reported the positive influence that psychosocial support had on their resilience capability, providing a strategy to implement for future learners. When referring to individual factors, only adaptive active cognitions were reported by GE learners. UG students mentioned that both adaptive and maladaptive active cognitions influenced their education capabilities. Maladaptive information processing biases were also reported by both UG and GE learners, highlighting the fact that all learners are susceptible to such biases. Finally, both maladaptive and adaptive executive control was reported by both groups of learners. These qualitative results therefore reveal routes through which UG participants could learn from mature age learners’ strategies with regards to cognitive management skills whilst acknowledging the fact that GE learners continued to show both adaptive and maladaptive tendencies with regards to information processing and executive control.

It is important to note that despite the significant correlations shown between resilience attitudes and burnout strategies and assessment outcomes, this does not indicate causation. Additionally, as the quantitative and qualitative results with regards to resilience and burnout strategies were self-reported by the participants, it is difficult to determine the impact of these strategies on their learning. Interestingly, GE students who came late into the program and had to “catch up” with the school leaver cohort didn’t seem to report being stressed. Although this study was conducted at a single institution in the pharmacy and pharmaceutical sciences realm, there is potential for application and expansion to other health professional degrees. The institution and health discipline was chosen because of the authors’ affiliations. Pharmacists are also known to leave their profession due to increased challenges in the workplace [21]. The scope of this research is also limited to those studying in the first year of their degree, with the potential for future research to expand this scope. Furthermore, this study employed an "opt-out" approach for participants, resulting in 100% participation from both the UG and GE cohorts. This could introduce a perceived power differential between students and researchers, who are likely faculty members, potentially influencing the response. Whilst the cohort sizes for the two groups are largely different, they both underwent the same assessments and same curriculum with the GE cohort completing the curriculum at an accelerated pace. Unfortunately, we are unable to dictate the class sizes as intake of GE is always much smaller than UG intake.

Whilst the importance of resilience in fostering students’ academic success is widely acknowledged, there is still a lack of implementation of resilience programs in higher education curricula. Mental health problems are known to be prevalent among higher education students before and after the pandemic [22, 23]. Universities now are faced with the responsibility to mitigate poor academic outcomes associated with mental health by enhancing students’ resilience to enable them to cope with the pressures of university [24, 25]. There also needs to be more comprehensive resilience data on differ types of students to be able to create support systems that suit today’s diverse cohort of students.

## Conclusion

Currently, there is still a need for resilience programs geared at academic success to be implemented in higher education. This study provides objective evidence of academic success coupled with exploration into the nuances of resilience amongst different student groups. It not only highlights the differing resilience development strategies and burnout coping mechanisms in emerging health professionals, but showcases juxtaposition of two different learner groups (UG and GE students) within a discipline. The cross-cohort facilitation of learning as in the discipline-specific strategies identified can help both groups develop resilience and inform future innovations. By comparing mature-age graduate students and younger-in-age undergraduate students, we identified a wider range of strategies and more positive attitudes to burnout in mature-age students. Health and clinical educators in university health degrees, clinical placements and clinical workplaces can develop effective training materials based on findings from this study to 1) assist undergraduate younger-age health students with developing resilience and 2) refine mature-age health students’ and practicing health professionals’ further resilience in today’s fast-paced clinical workplaces.

## Supplementary Information

Supplementary Material 1.

Supplementary Material 2.

Supplementary Material 3.

Supplementary Material 4.

## Data Availability

The dataset(s) supporting the conclusions of this article is(are) included within the article (and its additional file(s)).
