# Supplementary material for: The impact of resilience on academic performance with a focus on mature learners
Source: BMC Med Educ. 2024 Oct 7;24:1105. doi: 10.1186/s12909-024-06099-2 (PMC11460116; doi:10.1186/s12909-024-06099-2)
Supplement: Supplementary file 1 — Supplementary Material 1. [file 12909_2024_6099_MOESM1_ESM.docx]

**Resilience questionnaire**

For each of the following below, please select strongly agree, agree, neutral, disagree, strongly disagree

1. You received an ‘N’ on your most recent major assessment. The grades for your two other assessments were also lower than what you wanted. Please answer the following questions in relation to this prompt by imagining if you were in this same position.

I would begin to doubt my chances of success in the course

I would be disappointed

I would begin to think my chances of getting the job were poor

I would feel like everything was ruined and going wrong

I would try to think of new solutions

I would use my past successes to help motivate myself

I would set my own goals for achievements

I would seek encouragement from my family and friends

I would try to think about my strengths and weaknesses to help me work better

I would see the situation as a challenge

I would do my best to stop thinking negatively

I would see the situation as temporary

I would just give up

I would change my career plans

I would not change my long term goals and ambitions

1. Have you experienced burnout before? Please describe your experience e.g.how do you cope with burnout, how long did it last? What factors do you think increase your chances of experiencing burnout? [open text response answer]
2. What generally stresses you out? How do you relieve your stress? What coping methods do you use? [open text response answer]
3. What do you think the university should do / provide to help you improve your resilience? [open text response answer]
4. Write down a major thing that has hindered your learning over the past few weeks. This could be something specific to the course, but may also be something more general. [open text response answer]
5. Write down one strategy you could use in the future to reduce the impact of the above hindrance on your learning. Regardless of whether you can control the source of the hindrance, what is one thing that you can control that may reduce the negative impacts of that hindrance on your overall learning. [open text response answer]
