# Supplementary material for: The impact of resilience on academic performance with a focus on mature learners
Source: BMC Med Educ. 2024 Oct 7;24:1105. doi: 10.1186/s12909-024-06099-2 (PMC11460116; doi:10.1186/s12909-024-06099-2)
Supplement: Supplementary file 2 — Supplementary Material 2. [file 12909_2024_6099_MOESM2_ESM.docx]

**Interview Guide**

| - Firstly, we would like to discuss your results from the resilience survey and ask you to expand on your responses |
| --- |
| - Do you have goals in mind when you prepare for assessment tasks? |
| - How do you go about achieving these goals? |
| - What do you consider when getting ready to study/prepare for an assessment task? |
| - What motivates you to study/prepare for any assessment task? |
| - What are some demotivating factors and what do you do (if anything at all) to overcome these factors? |
| - Compare your learning experience in your prior degree to this degree (*for* ***grad entry*** *students only)* |
| - If you are really busy in both your studies and private life, how do you change your study techniques to be more efficient? |
| - How do you engage with other students in your degree when it comes to study? E.g. Study groups. |
| - How have you monitored your progress throughout your degree? |
| - How do you react to getting feedback from assessment tasks? |
| - Do you do anything with your feedback when you received? |
| - Can you predict whether or not you will do well on an assessment? What factors influence your predictions? |
