# Supplementary material for: The impact of resilience on academic performance with a focus on mature learners
Source: BMC Med Educ. 2024 Oct 7;24:1105. doi: 10.1186/s12909-024-06099-2 (PMC11460116; doi:10.1186/s12909-024-06099-2)
Supplement: Supplementary file 3 — Supplementary Material 3. [file 12909_2024_6099_MOESM3_ESM.docx]

**Summary of full quotes for environmental and individual factors for graduate entry and undergraduate students**

**Deductive Codes, Quotes and Frequencies for Graduate Entry Participants.**

| **Codes** | **Exemplar Quotes** | **Frequency N (%)**  **N = 9** |
| --- | --- | --- |
| Environmental Factors |  |  |
| Psychosocial Support | “My sister and I will debrief with my mom. And we just say I got this mark super happy with that, or got this mark, whatever, and I don't know, Mum's pretty good in asking, well, why do you think you know, is that good?” | 5 (55.6) |
| Receiving Feedback | “I'll definitely use feedback. I think it's really important for me, because I think I'll always find something that I don't know enough about” | 7 (77.8) |
| Workload | “I try to think about what I will do if tomorrow is my exam, like what I need to learn if I have to go to exam tomorrow when I'm really busy, and I don't have much time.” | 4 (44.4) |
| Individual Factors |  |  |
| Active Cognitions |  |  |
| Active Positive Cognition | “I'm not perfect, but I don't really have an issue with that.” | 3 (33.3) |
| Dysfunctional Cognition | *N/A* | 0 (0.0) |
| Executive Control |  |  |
| Adaptive | “I try to think about what I will do if tomorrow is my exam, like what I need to learn if I have to go to exam tomorrow when I'm really busy, and I don't have much time.” | 5 (55.6) |
| Maladaptive | “I guess I definitely will cut down the amount of time I spend on doing something which probably impacts on the quality of the way that I've done it for sure.” | 1 (11.1) |
| Information Processing Biases |  |  |
| Adaptive | “I take my mistakes very seriously, you know, it’ll still scar me, which is good because I remember.” | 1 (11.1) |
| Maladaptive | “I think I was too nervous to like stuff up in front of [my peers] or like to make a fool of myself if I didn't know what I was doing.” | 3 (33.3) |

**Deductive Codes, Quotes and Frequencies for Undergraduate Participants.**

| **Codes** | **Exemplar Quotes** | **Frequency N (%)**  **N = 13** |
| --- | --- | --- |
| Environmental Factors |  |  |
| Psychosocial Support | “If I don't understand it, I discuss it definitely with my friends, ask them to interpret it, and then, a further step from that is to consult my university” | 5 (38.5) |
| Receiving Feedback | “How are we supposed to learn if we don't know where we made our mistakes, so we don't repeat it in the future?” | 10 (76.9) |
| Workload | “I will make time, or I will find time to study in the nook like the nook and cranny.” | 6 (46.2) |
| Individual Factors |  |  |
| Active Cognitions |  |  |
| Active Positive Cognition | “I might not know everything, but I know at least a bit of everything, so I won't be completely lost.” | 1 (7.7) |
| Dysfunctional Cognition | “If it's a really low score that I didn't expect I'll get very sad.” | 1 (7.7) |
| Executive Control |  |  |
| Adaptive | “So if if there's like a motivation like I need to get this done, so I can progress on what I need to do tomorrow, cause this needs to get finished, I'd still go ahead and do it.” | 4 (30.8) |
| Maladaptive | “I thought, my biggest weakness with efficiency is less about study techniques. And just more in my head like getting myself to do stuff” | 2 (15.4) |
| Information Processing Biases |  |  |
| Adaptive | “I kind of make my own assumptions based off of the rubric of what I needed to improve on” | 1 (7.7) |
| Maladaptive | “I used to hate getting feedback from my assessment tasks, especially if I know I flunked it… I tried to avoid them as long as I could, because I feel like they criticizing me instead of like, giving me feedback.” | 2 (15.4) |
