# Supplementary material for: The impact of resilience on academic performance with a focus on mature learners
Source: BMC Med Educ. 2024 Oct 7;24:1105. doi: 10.1186/s12909-024-06099-2 (PMC11460116; doi:10.1186/s12909-024-06099-2)
Supplement: Supplementary file 4 — Supplementary Material 4. [file 12909_2024_6099_MOESM4_ESM.docx]

**Results from resilience survey regarding what the university can do to support resilience (Table 1), major hindrances to learning (Table 2) and naming one main strategy for the future (Table 3)**

**Table 1. What can the university do to support resilience?**

| **Undergraduate** | |
| --- | --- |
| Nothing more (current level of support is sufficient, students need to utilise more of what is offered, skills coaching is great) | 24 (19.7) |
| Being more accessible (easy to approach counsellors, utilise online format of education delivery, international students who feel alone in a different country, individual feedback, record workshops, receiving the ppt slides after lectures, learning intentions being made transparent, access upper classmates) | 24 (19.7) |
| Increased attention to wellbeing (frequent check-ins, words of encouragement, therapy, self-care cards, mindfulness, encouragement, self-care activities) | 14 (11.5) |
| Facilitating a supportive environment (support guidelines, after-school tutoring, return past papers to students, social events to break up studies, practice sessions before major assessments, adequate reminders, more engaging TA's, making hurdle assessments easier, resilience course, less work load, more time for break/study, spacing assessments) | 43 (35.2) |
| No further information/it is a personal problem (students should go through the pressure and develop resilience on their own) | 30 (24.6) |
| **Graduate Entry** | |
| Reduced reflective activities (reduced surveys) | 1 (3) |
| Increased activities (hamburger feedback, time management courses, help sessions on unsure topics, counselling, teaching soft skills early in career, i.e. dealing with burnout, listening to pharmacist alumni, stress relief-dog) | 13 (39.4) |
| Managing workload (increasing time between assessments and allocated preparation times) | 6 (18.2) |
| Increased accessibility of resources (having readily available answer sheets to corroborate, more practice exams) | 4 (12.1) |
| No further information/not the university's responsibility/nothing to change | 9 (27.3) |

**Table 2. Major Hindrances to learning**

| **Themes** | **UG** | **GE** |
| --- | --- | --- |
| Workload (overwhelming, stressing about previous exams, OSCE, fear of fail, hurdle assessments, study routine getting boring, connecting different topics together, failing unit) | 39 (29.8) | 14 (35.9) |
| Extraneous circumstances (personal, lack of motivation, family, work, tiredness, illness, travel to campus, financial difficulty, mental health, surgery, moving house, car crash) | 30 (22.9) | 12 (30.8) |
| Time management (procrastination, distractions, disproportionate division of attention, work/life balance, establishing study plan) | 47 (35.9) | 7 (17.9) |
| Institutional implications (timing of mid-semester break, marking disparity with TA's, repetition of tasks, mundane/difficult to understand lectures, simpler discovery, not being able to review previous exam, relating learned topics to real life) | 21 (16) | 4 (10.3) |
| No major hinderance (try to stay on top of tasks, no extra information) | 13 (9.9) | 6 (15.4) |

**Table 3. Name one strategy to use in the future**

| Themes | **UG** | **GE** |
| --- | --- | --- |
| Proactive planning (planning ahead, time management, prioritising understanding content over memorising, setting goals, reduce workload, self-learning through videos or external resources, discovering personalised learning strategies, breaking down the unit into smaller parts) | 65 (57) | 16 (48.5) |
| Self-care (being kind to yourself, be more confident, relax, eating, improving resilience, practising mindfulness, reward for completing tasks, de-stressing with family) | 22 (19.3) | 7 (21.2) |
| Study/Work/Life balance (paying attention to what matters in your life, increase study time) | 7 (6.1) | 3 (9.1) |
| Facilitating a productive environment of study (going to the library, removing distractions, increased focus, self-practice with a mirror, having friendly peers to help, expanding support network) | 18 (15.8) | 5 (15.2) |
| No individual plan (make suggestion about what university could do, just continue as is, sit down and study) | 14 (12.3) | 5 (15.2) |
